# *Mycobacterium smegmatis* Vaccine Vector Elicits CD4+ Th17 and CD8+ Tc17 T Cells With Therapeutic Potential to Infections With *Mycobacterium avium*

**DOI:** 10.3389/fimmu.2020.01116

**Published:** 2020-06-09

**Authors:** Nisha Kannan, Markus Haug, Magnus Steigedal, Trude Helen Flo

**Affiliations:** ^1^Center of Molecular Inflammation Research and Department of Clinical and Molecular Medicine, Norwegian University of Science and Technology (NTNU), Trondheim, Norway; ^2^Department of Infectious Diseases, St. Olavs University Hospital, Trondheim, Norway

**Keywords:** *Mycobacterium avium*, *Mycobacterium smegmatis*, ESX-3, infection, vaccine, CD8+ Tc17, inflammation

## Abstract

*Mycobacterium avium* (Mav) complex is increasingly reported to cause non-tuberculous infections in individuals with a compromised immune system. Treatment is complicated and no vaccines are available. Previous studies have shown some potential of using genetically modified *Mycobacterium smegmatis* (Msm) as a vaccine vector to tuberculosis since it is non-pathogenic and thus would be tolerated by immunocompromised individuals. In this study, we used a mutant strain of Msm disrupted in EspG_3_, a component of the ESX-3 secretion system. Infection of macrophages and dendritic cells with Msm Δ*espG*_3_ showed increased antigen presentation compared to cells infected with wild-type Msm. Vaccination of mice with Msm Δ*espG*_3_, expressing the Mav antigen MPT64, provided equal protection against Mav infection as the tuberculosis vaccine, *Mycobacterium bovis* BCG. However, upon challenge with Mav, we observed a high frequency of IL-17-producing CD4+ (Th17 cells) and CD8+ (Tc17 cells) T cells in mice vaccinated with Msm Δ*espG*_3_::*mpt64* that was not seen in BCG-vaccinated mice. Adoptive transfer of cells from Msm Δ*espG*_3_-vaccinated mice showed that cells from the T cell compartment contributed to protection from Mav infection. Further experiments revealed Tc17-enriched T cells did not provide prophylactic protection against subsequent Mav infection, but a therapeutic effect was observed when Tc17-enriched cells were transferred to mice already infected with Mav. These initial findings are important, as they suggest a previously unknown role of Tc17 cells in mycobacterial infections. Taken together, Msm Δ*espG*_3_ shows promise as a vaccine vector against Mav and possibly other (myco)bacterial infections.

## Introduction

*Mycobacterium avium* (Mav) is an opportunistic non-tuberculous mycobacterium (NTM) that mostly affects individuals with a compromised immune system (1–3). The drug regimen to eradicate Mav infections is arduous and often not successful and there are no vaccines available against Mav infections. However, a recent meta-analysis found evidence that vaccination with *Mycobacterium bovis* Bacillus Calmette–Guérin (BCG), the only available vaccine against *Mycobacterium tuberculosis* (Mtb), might exhibit cross-protection to infections with NTMs in immunocompetent individuals (4). Conversely, NTMs may exhibit anti-tuberculous resistance and also interfere with BCG vaccination (5). In addition to limited efficiency, an additional challenge with the BCG vaccine is that it is not well-tolerated by HIV-infected infants and other patients with a compromised immune system (6), leaving the people most vulnerable to mycobacterial infections without protection. Consequently, new vaccines with improved safety and efficacy profiles are needed to boost previously exposed or BCG-vaccinated individuals, and to combat emerging NTM infections for which we currently have no vaccines.

The development of new and improved vaccines against mycobacteria is challenging due to a lack of knowledge on the correlates of immune protection that could predict vaccine efficacy (7, 8). Adaptive immunity to Mav is considered to be mediated mainly by CD4+ T helper (Th) 1 cells (9, 10). In particular, the production of interferon (IFN) γ by CD4+ Th1 cells is important to control Mav infection, and mice genetically deficient in IFNγ have increased susceptibility to infection (9, 11). In addition to the Th1 response, mycobacterial infections also elicit a Th17 response (12–14). Early studies suggested that the IL-23/IL-17 axis was not critical for protection against tuberculosis (TB) in mice (15, 16). However, later studies in vaccinated mice provided evidence that Th17 cells may contribute to protection in mice that have been vaccinated with the Mtb antigen EsxA (13, 14). A role for the Th1/Th17 balance in Mav infection was suggested, wherein mice deficient for the transcription factor T-bet, critical for Th1 cell differentiation, showed a shift from Th1 toward Th17 responses and were more susceptible to Mav infection (17). Regarding the importance of CD8+ cytotoxic T (Tc) cells, experiments in mice deficient in CD8+ T cells indicated that Tc cells play a minor role in Mav infection (10, 18). However, as for Th cells, this could depend on the Tc cell subsets elicited. CD8+ T cells producing IL-17 (Tc17 cells) have been observed in pleural effusion of TB patients (19), and recently Loxton et al. (20) observed Tc17 cells in infants vaccinated with the strain BCG VPM1002. However, little is known about the functional role of Tc17 cells in mycobacterial infections.

Different approaches are used for the TB vaccine candidates currently under development and in clinical trials, either to replace BCG or to boost previously vaccinated or exposed individuals (7, 8). One approach is to improve safety and efficacy by engineering BCG or other mycobacteria to interfere with phagosome maturation and to express Mtb antigens. Another strategy pursues administration of Mtb antigens as subunit booster vaccines together with adjuvants. Prominent MTb antigens that have been included in TB vaccine candidates are EsxA, EsxH, and MPT64 which aid in MTb immune evasion (21–25). These proteins are secreted by various secretion systems like the early-secreted antigenic target secretion system (ESX, or type VII secretion system). Five ESX secretion systems (ESX-1 to ESX-5) are described within various mycobacterial species (26). ESX-3 is involved in iron uptake and is conserved across all mycobacterial species (27, 28). It has been shown that a modified strain of *Mycobacterium smegmatis* (Msm) in which the endogenous *esx-3* was exchanged with the Mtb *esx-3* locus, has potential as a vaccine against Mtb infections in mice (29). The vaccine strain elicited a pro-inflammatory milieu within mice and provided equal or superior protection, when compared to BCG, against subsequent Mtb challenge. However, it is not known if a Msm vaccine would provide protection against infections with NTM, such as bacteria of the *Mycobacterium avium* complex.

In this study, we created a Msm vaccine candidate strain that is deficient in the ESX-3 secretion-associated chaperone protein EspG_3_. We and others have previously shown that EspG_3_ is an important component for ESX-3 function (27, 30, 31), and that Msm Δ*espG*_3_ functionally resembles the Msm Δ*esx-3* mutant used by Sweeney et al. (29). Our results show that Msm Δ*espG*_3_ was more efficient than Msm wild-type (WT) in activating CD4+ T cells specific to a model antigen, ovalbumin (OVA). Vaccination of mice with Msm Δ*espG*_3_ expressing a Mav antigen, MPT64 (Msm Δ*espG*_3_::*mpt64*), offered similar protection as BCG against Mav infection. A strong induction of IL-17-producing CD4+ and CD8+ T cells in response to Mav infection in the Msm Δ*espG*_3_::*mpt64*-vaccinated mice, revealing a possible role of IL-17-producing T cells in controlling Mav infection.

## Materials and Methods

### Mycobacterial Strains

*M. avium 104, M. bovis (BCG, Inter Vax), M. smegmatis* mc^2^155 (Msm), Msm Δ*espG*_3_ (27), and Msm Δ*espG*_3_::*pDE43MPT64* (Msm Δ*espG*_3_::*mpt64*) were used for the experiments performed. The Msm Δ*espG*_3_::*mpt64* was generated from Msm Δ*espG*_3_ by introducing *mpt64 (MAV_4130)* under a constitutive promoter p750 into the mycobacterial shuttle vector pDE43 (32). The bacteria were grown on 7H10 Middlebrook (Difco/Becton Dickinson) plates supplemented with 10% ADC (Difco/Becton Dickinson). Subsequently, single colonies were transferred to 7H9 Middlebrook (Difco/Becton Dickinson) liquid medium supplemented with 10% Middlebrook ADC Enrichment (Difco/Becton Dickinson). Cultures were grown to logarithmic phase (OD_600_ 0.5–0.6) at 37°C, then pelleted down and suspended in phosphate-buffered saline (PBS). Bacteria were sonicated and passed through a 25 × 5/8 G needle to obtain single cell suspensions, which were further used in *in vitro* or *in vivo* infections.

### Cell Culture

Bone marrow cells were isolated from femurs of C57BL/6 mice. Red blood cells were lysed (RBC lysis buffer, eBioscience) before cells were resuspended in RPMI-1640 medium (Sigma) supplemented with 10% fetal calf serum (Gibco). To differentiate cells toward bone marrow-derived macrophages (BMDMs), the medium was supplemented with 20% supernatant from L-929 cells for 4–5 days. Ten nanograms per milliliters of mouse Granulocyte-macrophage colony-stimulating factor (Stem cell technologies) was added for 6–7 days to differentiate cells primarily toward bone marrow-derived dendritic cells (BMDCs), though the resulting cell population has been described to also contain other myeloid cell types (33).

CD4+ and CD8+ T cell hybridoma lines MF2.2D9 and RF33.7 (kind gift of Kenneth Rock, University of Massachusetts) were cultured in RPMI-1640 medium containing 10% fetal calf serum, 50 μM β-mercaptoethanol (Sigma), 25mM HEPES (Gibco), and 10μg/ml ciprofloxacin (Cell-Gro). IL-2 dependent HT-2 reporter cells (ECACC) were cultured in RPMI-1640 medium (Sigma) supplemented with 10% fetal calf serum, 50 μM β-mercaptoethanol, 1% sodium pyruvate (Sigma), 1 ng/ml recombinant interleukin-2 (RnDSystems), and 10 μg/ml ciprofloxacin.

### *In vitro* Infection of BMDMs and BMDCs

BMDMs or BMDCs were seeded in a 96 well-plate (50,000 cells/per well in triplicate) in 200 μl RPMI-1640 medium (Sigma) supplemented with 10% fetal calf serum and cultivated overnight. Cells were infected with WT Msm and Msm Δ*espG*_3_ at a multiplicity of infection (MOI) of 10. Infection was performed for 2 h. To remove and kill extracellular bacteria, the cells were washed with Hanks balanced salt solution (Sigma), then incubated for 30 min in medium containing 10 μg/ml gentamicin (Sanofi) and washed again. Treatment of cells with 10 μg/ml gentamicin for 30 min prevented cells from overgrowth with extracellular bacteria and did not affect cell health. Directly after infection as well as 4, 10, and 24 h post-infection, cells were lysed with phosphate buffered saline (Sigma) containing 0.02% Triton X (Sigma). The lysate was plated in serial dilutions on 7H10 Middlebrook plates to quantify colony forming units (CFU). To assess major histocompatibility complex (MHC) class II and CD86 expression as well as nitric oxide production in Msm infected cells, BMDMs were seeded in 24 well-plates (500,000 cells/per well) and infected for 24 h as described above. To quantify nitric oxide production, cells were incubated for the last 30 min with 1 mM 4-amino-5-methylamino-2′,7′-difluororescein diacetate (DAF-FM diacetate, Invitrogen), control cells samples were treated with lipopolysaccharide (100 ng/ml, Sigma). To assess MHC class II and CD86 expression, cells were removed from the plates, treated with Fc block (anti-CD16/CD32, eBioscience) and stained with fluorescent monoclonal antibodies to MHC class II (I-Ab Alexa Fluor 488, BioLegend) and CD86 (Phycoerythrin, BD Biosciences). Nitric oxide production and MHC class II and CD86 expression was analyzed by flow cytometry on a BD LSR II flow cytometer (BD Biosciences) and analyzed with FlowJo_v.10 (FlowJo, LLC). GraphPad Prism 8 software (GraphPad Software, Inc.) was used to perform statistical analyses.

### Plasmid Constructs

For the overexpression of *mpt64*, we used Gateway technology (Sigma) to clone our inserts. Entry clones for *mpt64* and p750, the constitutive promoter, were made following a PCR with the following primers and a subsequent Gateway BP reaction.

B1-p750 promoter: 5′GGGGACAAGTTTGTACAAAAAAGCAGGCTGGCTCTGACTTGAGCGTCGATTT3′.

B5r-p750 promoter:

5′ GGGGACAACTTTTGTATACAAAGTTGTCTCAAAGGCGGTAATACGG 3′.

B5-*mpt64:* 5′GGGGACAACTTTGTATACAAAAGTTGTAATGCGCAGTTTCAGCGTGGCAGCG3′.

B2-*mpt64:* 5′GGGGACCACTTTGTACAAGAAAGCTGGGTAGGCGAGCATCGGGTCGATCGCGGAA 3′.

pDE43 (kindly provided by D. Schnappinger, Weill Cornell, NY) is a Gateway compatible destination vector that can be used in mycobacteria. Entry clones *mpt64*, p750, and pDE43 were fused to generate clones containing *mpt64* under a constitutive promoter p750. Gateway reactions were performed as specified by the manufacturer. For over-expression of truncated ovalbumin (OVA), PCR primers were designed that amplified the region containing both the MHC class I antigenic epitope OVA_257−264_ (SIINFEKL) and the MHC class II antigenic epitope OVA_323−339_ (ISQAVHAAHAEINEAGR). Primer sequences: Truncated OVA forward primer 5′GGAATTCCATATGGGGATCCTGGAGCTTCCA 3′, truncated OVA reverse primer 5′ ACATGCATGCCTAGTCTTCAGAGACGCT 3′. Plasmids containing constitutive promoter pMV261 were used. Both the insert and plasmid were digested with NdeI and SphI (both New England Biolabs, Inc.). Clones generated were subsequently sequenced.

### qPCR

RNA was quantified from both WT and Δ*espG*_3_ Msm either overexpressing truncated OVA protein or the Mav MPT64 protein (Supplementary Figure 1). Bacteria grown to exponential phase were pelleted down and resuspended in 600 μl Trizol (Invitrogen). The resuspended bacteria were added to tubes containing 0.1 mm beads (Bertin Technology) and bead-beated twice for 2 min with a FastPrep-24 instrument (MP biosystems, 4.0 M/S, 30 s ON and 30 s OFF). One-hundred and fifty microliters of chloroform was added to the supernatant after bead-beating. The aqueous phase containing RNA was carefully transferred into 1.5 ml RNase free Eppendorf tubes and 300 μl cold isopropanol (Teknisk) was added and kept at −20°C for 15 min. RNA was isolated (Qiagen RNA purification Kit) and subsequently reverse transcribed to cDNA (Applied Biosystems). Quantitative real-time PCR was performed in duplicates on a StepOnePlus qPCR System (Applied Biosystems) with primers to truncated OVA (forward primer 5′GTTGGTGCTGTTGCCTGATG3′, reverse primer 5′ CTCTGCTGAGGAGATGCCAG 3′) and Mav *mpt64* (forward primer 5′GATCAGCCCTACCAGCTGAC 3′, reverse primer 5′ TTCTGGACGACCTTGAGCAC 3′). RNA polymerase sigma factor SigA expression was analyzed as reference for normalization of qPCR data.

### *In vitro* Antigen Presentation and T Cell Activation Assay

BMDCs were generated as described and seeded in 24 well-plates (500,000 cells per well) for *in vitro* analysis of MHC class I antigen presentation experiments or in 96 well-plates (50,000 cells per well) for *in vitro* analysis of CD4+ and CD8+ T cell cell activation. The cells were infected with WT Msm and Msm Δ*espG*_3_ overexpressing truncated OVA or the respective empty vector control strains at an MOI of 10. Treatment with full-length chicken OVA protein (225 μM, Sigma) plus lipopolysaccharide (100 ng/ml, Sigma) was carried out as positive control for antigen presentation. After Msm infection for 2 h, cells were washed and extracellular bacteria eliminated (30 min, 10 μg/ml gentamicin).

Analysis of OVA-specific MHC class I antigen presentation: 24 h post-infection, BMDCs were removed from the plate, treated with Fc block (anti-CD16/CD32, eBioscience) and stained with fluorescent monoclonal antibody to CD11c (Fluorescein isothiocyanate, BD Biosciences) and OVA_257−264_ (SIINFEKL) peptide in complex with MHC class I (H-2Kb/SIINFEKL Phycoerythrin, eBioscience). Flow cytometry was performed on a BD LSR II flow cytometer (BD Biosciences) and analyzed with FlowJo_v.10 (FlowJo, LLC). GraphPad Prism 8 software (GraphPad Software, Inc) was used to perform statistical analyses.

Analysis of CD4+ and CD8+ T cell activation: Directly after infection, BMDCs were washed and subsequently overlaid with 1 × 10^5^ OVA-specific T cell hybridoma cells per well and incubated overnight at 37°C. RF33.7 cells were used to detect MHC class I presentation of the OVA_257−264_ (SIINFEKL) epitope and MF2.2D9 cell line to detect MHC class II presentation of the OVA_323−339_ (ISQAVHAAHAEINEAGR) epitope, as described in Moura Rosa et al. (34) and Gopalakrishnan et al. (35). Supernatants from the BMDC:T cell hybridoma co-culture were collected and production of bioactive cytokines from activated RF33.70 or MF2.2D9 hybridoma cells was quantified in a bio-assay (36). For this, IL-2 dependent HT-2 cells (1 × 10^4^ cells/well) were cultured overnight in 50% supernatant from BMDC:T cell hybridoma co-culture before proliferation of HT-2 cells was analyzed using the CellTiter 96 Aqueous One Cell Proliferation Assay (Promega).

### Mouse Vaccination and Infection

All protocols on animal work were approved by the Norwegian National Animal Research Authorities and carried out in accordance with institutional guidelines, national legislation, and the Directive of the European Convention for the protection of animals used for scientific purposes. Six to eight week old C57BL/6 mice were bred in house at the Comparative Medicine Core Facility (CoMed) at NTNU and used for vaccination and infection experiments.

Subcutaneous vaccinations were performed with Msm Δ*espG*_3_::*mpt64* and BCG. 1 × 10^7^ bacteria were injected in 100 μl PBS, sham-vaccinated mice received 100 μl of PBS. Msm Δ*espG*_3_::*mpt64* vaccination was performed twice with a 15 day interval, BCG vaccination was performed with a single vaccination dose (1 × 10^7^ bacteria, **Figure 2A**). An aliquot of the inoculum was plated in serial dilution on Middlebrook 7H10 plates to verify the bacteria number. Thirty days post the first vaccination, mice were challenged with the virulent Mav strain 104 (**Figure 2A**). Infection was performed by intraperitoneal injection of log-phase Mav strain 104 (1 × 10^9^ CFUs in 200 μl PBS per mouse). Thirty days after infection, mice were killed, and spleen and liver were collected for CFU counting and T cell analysis. Bacterial load was measured by plating serial dilutions of organ homogenates (spleen, liver) on Middlebrook 7H10 plates.

### Mycobacteria-Specific T Cell Cytokine Production and Memory T Cell Analysis

Mav-specific effector T cell cytokine production was analyzed from splenocytes of mice. Splenocytes from mice were isolated 30 days post Mav infection, stimulated overnight with Mav (MOI 3:1) and prepared for flow cytometry as previously described (37). Protein transport inhibitor cocktail (eBioscience) was added for the last 4 h of stimulation. Surface antigens were characterized by staining with fluorescence-labeled monoclonal antibodies against CD3 (Fluorescein isothiocyanate), CD4 (Brilliant Violet 605), CD8 (Brilliant Violet 785, all BioLegend). After fixation and permeabilization (eBioscience Intracellular Fixation and Permeabilization Buffer Set), intracellular cytokine production was analyzed by staining with fluorescent monoclonal antibodies to IFNγ (Phycoerythrin), TNFα (Allophycocyanin) and IL-17 (PE/Cy7, all Biolegend). For memory T cell phenotype analysis, unstimulated splenocytes were stained as described above for CD3, CD4 and CD8. Additionally, cells were stained with monoclonal antibodies against CD44 (Alexa Fluor 700) and CD62L (Brilliant Violet 510, both from Biolegend). Flow cytometry was performed on a BD LSR II flow cytometer (BD Biosciences) and data subsequently analyzed with FlowJo_v.10 (FlowJo, LLC). Statistical analyses were performed with GraphPad Prism 8 software (GraphPad Software, Inc).

### Adoptive Transfer

A timeline of the procedures for the adoptive transfer experiments can be found in **Figure 4**. Adoptive transfer experiments were performed with a lower Mav infection dose (1 × 10^7^ CFUs) compared to the initial vaccination experiments (1 × 10^9^ CFUs). Adoptive transfer of total CD3+ T cells from vaccinated mice was solely tested in a therapeutic setting (adoptive CD3+ transfer 15 days post Mav infection of recipient mice). Adoptive transfer of Tc17-enriched and Tc17-depleted T cells was assessed in a prophylactic (1 day prior to Mav infection) or therapeutic setting (15 days post Mav infection).

Vaccination of mice for adoptive transfer: Donor mice for adoptive cell transfer were vaccinated with Msm Δ*espG*_3_::*mpt64* or sham-vaccinated as described above. On day 28, Msm Δ*espG*_3_::*mpt64* and sham-vaccinated mice were infected with Mav strain 104 (1 × 10^7^ in 200 μl PBS) to allow generation of fully active Mav-specific effector T cells. Two days later, donor mice were killed, and spleens harvested for isolation of CD3+, CD8+IL-17–, and CD8+IL-17+ T cells.

Isolation of T cell fractions for adoptive transfer: Total CD3+ T cells were purified from spleens of Msm Δ*espG*_3_::*mpt64* or sham-vaccinated mice using the Dynabeads Untouched Mouse T Cell kit (Thermo Fisher). For Tc17-enrichment or Tc17-depletion, untouched CD8+ T cells were isolated from spleens of Msm Δ*espG*_3_::*mpt64*-vaccinated mice (CD8a+ T cell isolation kit, Miltenyi Biotec). CD8+IL-17– and CD8+IL-17+ T cell fractions were enriched from CD8+ T cells using the mouse IL-17 Secretion Assay Cell Enrichment and Detection Kit according to the manufacturer's protocol (Miltenyi Biotec). The purity of adoptively transferred T cell fractions was assessed by flow cytometry and is exemplified in Supplementary Figure 6. CD3+ T cells for adoptive transfer experiments had a purity of >90%, Tc17-depleted cells contained <1% and and Tc17-enriched T cell fractions >10% of IL-17-producing CD8+ T cells.

Adoptive transfer procedures: In therapeutic setup experiments, total CD3+ T cells or Tc17-enriched or -depleted T cell fractions (~ 1.4 × 10^6^ CD3+, ~ 1.5 × 10^5^ CD8+IL-17–, or ~ 1.5 × 10^5^ CD8+IL-17+ enriched T cells in 100 μl PBS) from donor mice were intravenously injected into recipient mice 15 days post-infection of recipient mice with 1 × 10^7^ CFUs Mav strain 104. In prophylactic setup experiments, Tc17-depleted or Tc17-enriched T cell fractions (~4 × 10^5^ CD8+IL-17– or ~4 × 10^5^ CD8+IL-17+ T cells in 100 μl PBS) were intravenously injected into recipient mice. The following day, recipient mice were infected with 1 × 10^7^ CFUs Mav strain 104. In both, the therapeutic and prophylactic experimental setup, bacterial load (liver and spleen) and T cell effector responses (spleen) of recipient mice were analyzed 30 days post Mav infection as described above.

## Results

### EspG_3_-Deficient Msm Shows Reduced Survival in Antigen Presenting Cells and Increased T Cell Activation Compared to Wild-Type Msm

**Figure 1:**
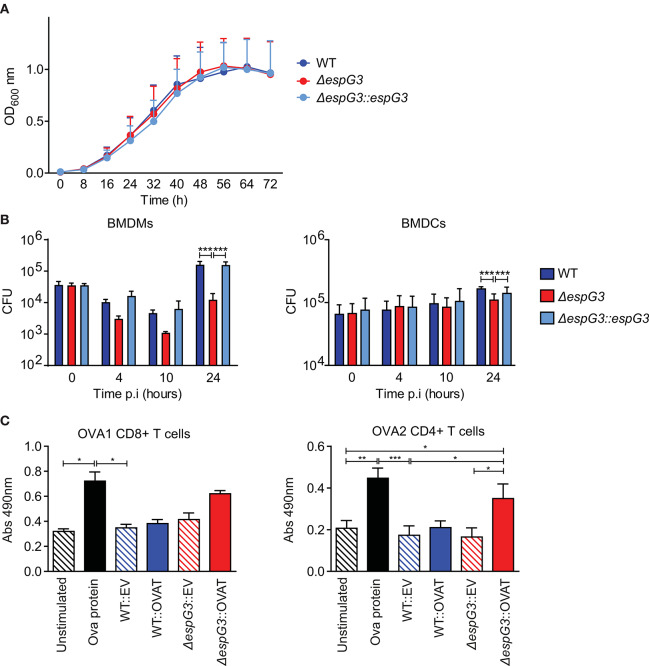
Analysis of Msm *in vitro* growth, survival in APCs, and antigen presentation. **(A)** *In vitro* growth curve assays for Msm WT, Δ*espG*_3_, and complemented Δ*espG*_3_::*espG*_3_. Strains were grown in7H9 medium, from a starting OD_600_ of 0.02 for 72 h. Growth curves were performed in triplicate, results are presented as means + SEM. **(B)** Intracellular replication of Msm strains in murine BMDMs (left) and BMDCs (right). BMDMs and BMDCs were infected with Msm WT, Δ*espG*_3_, and complemented Δ*espG*_3_::*espG*_3_ at a MOI of 10. CFU counts from lysed BMDMs and BMDCs were determined at 0, 4, 10, and 24 h post-infection (p.i.). Results display means + SEM from three (BMDMs) or four (BMDCs) experiments, statistical analysis was performed by repeated measures two-way ANOVA, Tukey post-test; Significance level: ***p < 0.001. **(C)** Analysis of antigen-presentation and T cell activation from Msm-infected APCs. BMDCs were infected with Msm WT or Δ*espG*_3_ either overexpressing truncated ovalbumin protein (WT::OVAT and Δ*espG*_3_::OVAT) or transformed with the empty destination vector (EV) pDE43 alone (WT::EV and Δ*espG*_3_::OVAT). BMDCs stimulated with OVA protein (225 μM) were used as positive control, unstimulated BMDCs as negative control. Infected BMDCs were overlaid with either OVA1 or OVA2 specific CD4+ and CD8+ hybridoma T cells. Production of bioactive cytokines from activated CD4+ and CD8+ T cells was measured in a bio-assay analyzing growth of the IL-2 dependent cell line HT-2 (Abs 490nm). Results are displayed as means + SEM from three independent experiments; repeated measures one-way ANOVA with Tukey post-test; significance level: *p < 0.05, **p < 0.01, ***p < 0.001.

It has previously been shown that vaccination of mice with Msm in which the endogenous *esx-3* locus is complemented with Mtb *esx-3*, promotes protective immunity against Mtb and thus has potential as a novel vaccine vector (29). We wanted to investigate if Msm deficient in the ESX-3 component, EspG_3_, by itself was superior compared to WT Msm in enabling antigen presenting cells (APCs) to present antigens produced within the bacterium. First, we confirmed that Msm Δ*espG*_3_, Msm WT, and the complemented Msm strain (Δ*espG*_3_::*espG*_3_) had comparable growth rates when cultivated *in vitro* (Figure 1A). Subsequently, we assessed survival of the Msm strains within APCs. Bone marrow-derived dendritic cells (BMDCs) and bone marrow-derived macrophages (BMDMs) were infected with WT Msm, Msm Δ*espG*_3_, and the reconstituted Msm Δ*espG*_3_::*espG*_3_. Twenty-four hours of post-infection, the bacterial load of Msm Δ*espG*_3_ was one log (90%) lower compared to Msm WT or reconstituted Msm Δ*espG3::espG3* in BMDMs (Figure 1B, left graph). In BMDCs, the difference was less pronounced with 35% lower CFUs of Msm Δ*espG3* compared to Msm WT and the reconstituted strain (Figure 1B, right graph). This reduced survival could result from restricted intracellular growth or from improved detection and clearance of Msm Δ*espG*_3_ compared to Msm WT, which could impact antigen presentation in different ways. We thus addressed activation status and nitric oxide production in BMDMs post Msm infection (Supplementary Figure 1A). Infection with all three Msm strains increased the surface expression of MHC class II on BMDMs. But while infection with Msm WT and Msm Δ*espG*_3_::*espG*_3_ strongly increased expression levels of the T cell co-stimulatory molecule CD86, the Msm Δ*espG*_3_ strain failed to do so. None of the three Msm strains induced nitric oxide production in infected BMDMs.

To further assess the effectiveness of Msm Δ*espG*_3_ as an antigen-presenting vehicle, we introduced a truncated ovalbumin construct encoding the C57BL/6 mouse MHC class I epitope OVA_257−264_ (OVA1) and the MHC class II epitope OVA_323−339_ (OVA2) in Msm WT and Msm Δ*espG*_3_. Only Msm Δ*espG*_3_ expressing the OVA2 epitope significantly increased activation of OVA2-specific CD4+ T hybridoma cells by infected BMDCs (Figure 1C, right). In contrast, OVA1-specific CD8+ T hybridoma cells were not significantly activated by BMDCs infected with the OVA1-expressing Msm Δ*espG3*. Infection with Msm WT did not increase CD4+ or CD8+ T cell activation, independent of OVA expression. We further directly assessed antigen presentation of OVA1 peptides via MHC class I on BMDCs by antibody staining of MHC class I/OVA_257−264_ complexes (Supplementary Figure 1C). OVA1 peptides were presented in BMDCs infected with OVA1 expressing Msm strains. However, we did not observe significant differences in MHC class I-restricted antigen presentation between the OVA1-expressing Msm Δ*espG3* and WT strain. Taken together, the findings of reduced Msm Δ*espG*_3_ survival in APCs and increased CD4+ T cell activation suggest that Msm Δ*espG*_3_ could be a better vaccine vector than Msm WT.

### Msm Vaccination Protects Against Mav Infection

**Figure 2:**
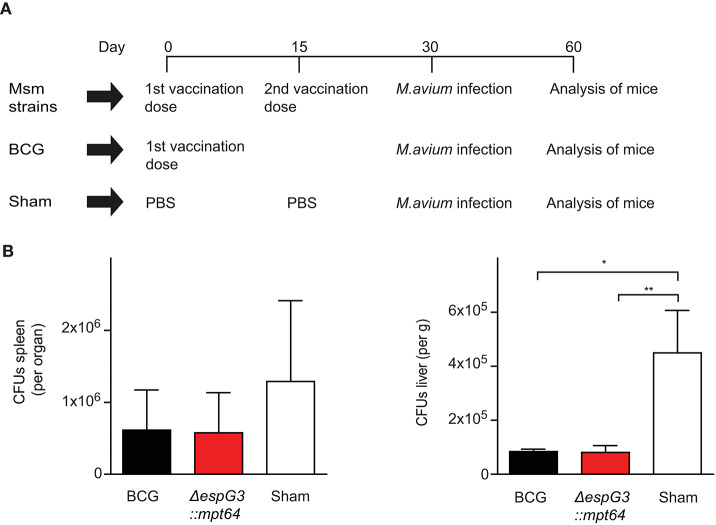
Effect of Msm vaccination on Mav infection is similar to the effect of BCG. **(A)** Vaccination protocol. C57BL/6 mice received either two subcutaneous vaccinations of 1 × 10^7^ CFUs Msm at a 15-day interval or one dose of *M. bovis* BCG; sham-vaccinated mice received PBS. Msm WT, Msm Δ*espG*_3_, and Msm Δ*espG*_3_::*mpt64* strains were used for Msm vaccination. Thirty days post the first vaccination, mice were infected *intraperitoneally* with 1 × 10^9^ CFUs Mav strain 104. On day 30 post Mav infection, livers and spleens were harvested for analysis of bacterial load and Mav-specific T cell responses. **(B)** Mav bacterial load (CFUs) was quantified in spleen (left) and liver (right) of mice vaccinated with BCG, Msm Δ*espG*_3_::*mpt64*, or sham-vaccinated mice 30 days post Mav infection. Results represent mean + SEM of three vaccination experiments with four mice per group; repeated measures one-way ANOVA, Tukey post-test; significance levels: *p < 0.05; **p < 0.01.

To evaluate the effectiveness of Msm Δ*espG*_3_ as a vaccine vector *in vivo*, we replaced the OVA test-antigens with the Mav antigen MPT64, creating the strain Msm Δ*espG*_3_::*mtp64*. MPT64 is an immunodominant secretory antigen (38) and the Mtb homolog has been used as antigen in vaccine studies against TB (23–25). We vaccinated mice subcutaneously with Msm Δ*espG*_3_::*mtp64* and included a booster vaccination at day 15, since Msm Δ*espG*_3_ is rapidly cleared from mice (29). BCG vaccination, which is commonly administered in a single dose, was performed for comparison. Sham-vaccinated mice received PBS (vaccination scheme shown in Figure 2A). Thirty days post the first vaccination, mice were challenged with Mav strain 104, and CFUs in liver and spleen were quantified 30 days after challenge (Figure 2B). Organ bacterial load was significantly lower in the liver of both BCG and Msm Δ*espG*_3_::*mtp64*-vaccinated mice compared to sham-vaccinated mice. In spleens, reduction of organ bacterial load was not statistically significant in any vaccination group compared to sham-vaccinated mice.

To address whether the Δ*espG*_3_ deletion or MPT64 expression in Msm are required for the protective effect on Mav infection, we vaccinated mice with Msm WT, Msm Δ*espG*_3_ or Msm Δ*espG*_3_::*mtp64* before challenge with Mav strain 104 (Supplementary Figure 2A). Organ bacterial load in spleens was reduced in all Msm vaccination groups. The reduction was found statistically significant for mice receiving Msm WT and Msm Δ*espG*_3_ vaccination, but not for BCG or Msm Δ*espG*_3_::*mtp64*-vaccinated mice. In the liver, organ bacterial load was significantly lower in all vaccination groups compared to sham-vaccinated mice. Hence, we concluded that Msm vaccination has a protective effect on Mav infection in mice and that the degree of protection conferred by WT, Msm Δ*espG*_3_ as well as Msm Δ*espG*_3_::*mtp64* was comparable to that of BCG. Overexpression of the Mav antigen MPT64 in the Msm Δ*espG*_3_ mutant did not provide an additional effect on protection of mice from Mav infection.

### Mice Immunized With Msm *ΔespG_3_*::*mtp64* Elicit a Strong Th17 and Tc17 Response

Mycobacteria-specific T cells producing effector cytokines are a central element for successful control of Mav infection (1, 37, 39). To evaluate the effector T cell subset composition after Msm Δ*espG*_3_::*mtp64* or BCG vaccination, splenocytes of vaccinated mice were isolated 30 days post Mav challenge and stimulated *in vitro* overnight with Mav strain 104. CD4+ and CD8+ T cells were analyzed for IFNγ, TNFα, and IL-17 effector cytokine production (gating strategy in Supplementary Figure 3, staining examples from vaccinated mice in Supplementary Figure 4).

**Figure 3:**
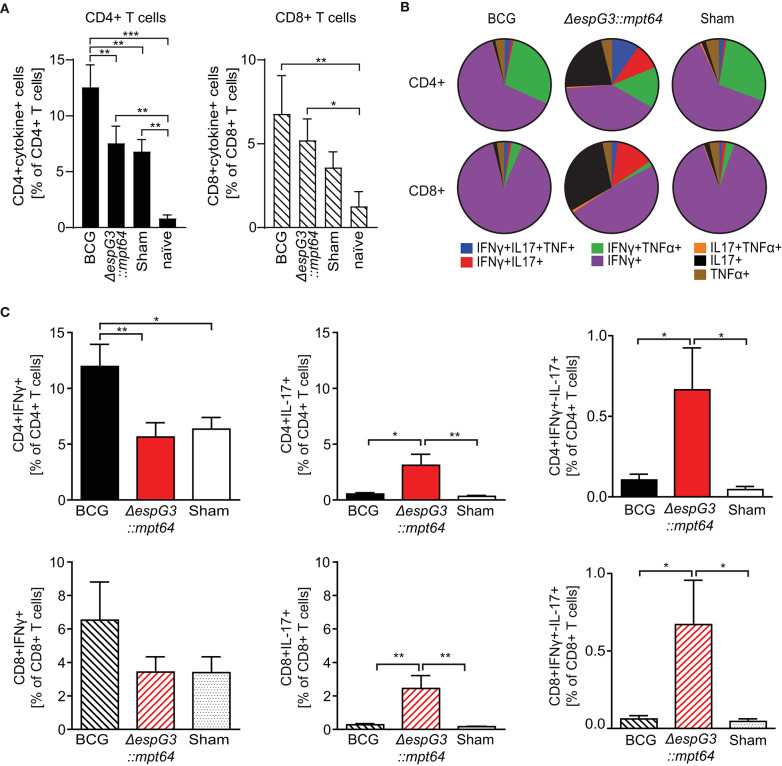
Msm Δ*espG*_3_::*mpt64* vaccination results in increased frequencies of IL-17-producing CD4+ and CD8+ T cells. C57BL/6 mice were vaccinated with BCG, Msm Δ*espG*_3_::*mpt64*, or sham-vaccinated and infected with Mav as described in Figure 2. Thirty days post Mav challenge, splenocytes were isolated and stimulated overnight with Mav. Mav-specific CD4+ and CD8+ effector cytokine production was analyzed by intracellular flow cytometry. Cells were gated as described in Supplementary Figure 3. Data from three individual vaccination experiments with four mice in each vaccination group were combined to generate the graphs in panels **(A–C)**. **(A)** Total Mav-specific effector T cell cytokine responses. Frequencies of CD4+ and CD8+ T cells that produce any of the effector cytokines IFNγ, IL-17, or TNFα upon Mav stimulation were identified. Results are displayed as % of CD4+ and CD8+ T cells. Unvaccinated and uninfected naïve mice were used as controls. Bars represent means + SEM. **(B)** Multifunctionality of Mav-specific T cells. The subsets of cytokine-producing CD4+ and CD8+ T cells identified in panel **(A)** were further analyzed by Boolean gating for simultaneous production of one or more effector cytokines. Mean values of single, double, and triple cytokine-producing CD4+ (top row) and CD8+ T cells (lower row) are displayed as pie charts. **(C)** Subset analysis of IFNγ- and IL-17-producing Mav-specific CD4+ and CD8+ T cells. Frequencies of IFNγ-(left), IL-17-(middle), and IFNγ/IL-17-(right) producing cells were identified from the total cytokine-positive subsets identified in panel **(A)**, irrespective of their TNFα production status. Data is displayed as bar charts representing means + SEM. Shown are percentages of effector cytokine-producing T cells in the CD4+ (top row) and CD8+ (lower row) gate. Bars represent means + SEM. Statistical analyses in panels **(A,C)** were performed by repeated measures one-way ANOVA with Tukey post-test; Significance levels *p < 0.05; **p < 0.01; ***p < 0.001.

We first examined if the total frequency of CD4+ and CD8+ T cells producing effector cytokines (IFNγ, TNFα, or IL-17) in response to Mav stimulation varied between the different vaccines in our study (Figure 3A). We observed Mav-specific effector cytokine production in about 13% of CD4+ T cells from BCG-vaccinated mice, compared to about 7% in both Msm Δ*espG*_3_::*mtp64*- and sham-vaccinated mice. The frequencies of cytokine-producing CD8+ T cells were higher in mice vaccinated with BCG and Msm Δ*espG*_3_::*mtp64* compared to sham-vaccinated mice (7 vs. 5 and 4%, respectively). We further addressed multifunctionality of Mav-specific CD4+ and CD8+ T cells by analyzing the distribution of single-, double- and triple-producers of the effector cytokines IFNγ, TNFα, or IL-17 within the total population of cytokine-producing CD4+ and CD8+ T cells (Figure 3B). A difference could indicate which effector T cell subsets and mechanisms are involved in controlling the Mav infection. The majority of the CD4+ T cells in BCG- and sham-vaccinated mice were typical Th1 cells producing IFNγ or IFNγ/TNFα (combined more than 80% of cytokine-producing cells, Figure 3B upper charts). In contrast, increased frequencies of IL-17 or IFNγ/IL-17 (combined ~35% of cytokine-producing cells) expressing CD4+ cells were seen in Msm Δ*espG*_3_::*mtp64*-vaccinated mice. A small fraction of triple-producing CD4+ cells (IFNγ, TNFα, and IL-17) was seen in all groups and most in Msm Δ*espG*_3_::*mtp64*-vaccinated mice (10% of the total cytokine-producing CD4+ cells compared to 2% in BCG and 2% in sham-vaccinated mice).

The majority of Mav-specific CD8+ effector T cells produced IFNγ in all vaccination groups; fewer IFNγ/TNFα double cytokine-producing cells were found compared to CD4+ T cells (Figure 3B, lower charts). As with CD4+ T cells, we similarly observed that BCG mainly induced IFNγ producing effector CD8+ T cells, whereas Msm Δ*espG*_3_::*mtp64* induced increased frequencies of IL-17-producing CD8+ effector T cells. In Msm Δ*espG*_3_::*mtp64*-vaccinated mice, about 30% of the cytokine-producing CD8+ T cells were IL-17 single producers, 50% IFNγ single producers, and 13% IFNγ/IL-17 double producers. Conversely, about 90% of the CD8+ T cells from BCG-vaccinated mice produced IFNγ only, and <1% were IL-17 single producers or IFNγ/IL-17 double producers, respectively. As with the CD4+ T cells, the CD8+ T cell subset distribution in sham-vaccinated mice resembled what was seen in BCG-vaccinated mice.

Combining the frequencies of total cytokine-producing CD4+ and CD8+ T cells (Figure 3A) with the effector T cell subset distribution (Figure 3B), a significantly higher frequency of IFNγ-producing CD4+ Th1 cells as well as IFNγ-producing CD8+ T cells (Tc1 cells) were found in BCG-vaccinated mice compared to Msm Δ*espG*_3_::*mtp64-* and sham-vaccinated mice (Figure 3C, left). In contrast, more IL-17-producing CD4+ Th17 and CD8+ Tc17 cells were seen in mice vaccinated with Msm Δ*espG*_3_::*mtp64* compared to BCG- or sham-vaccinated mice (Figure 3C, middle). In addition, when compared to BCG- and sham-vaccinated mice, there were increased frequencies of IFNγ/IL-17 double cytokine-producing CD4+ and CD8+ T cells in Msm Δ*espG*_3_::*mtp64*-vaccinated mice, though frequencies of these cells were relatively low (<1%, Figure 3C, right).

Finally, memory cell generation was assessed as a protective correlate of the vaccines (Supplementary Figure 5). Msm Δ*espG*_3_::*mtp64* induced significantly more CD4+ T cells with a T central memory phenotype (CD44^hi^CD62L^hi^) than BCG, no significant difference was observed in CD8+ T central memory cells between Msm Δ*espG*_3_::*mtp64* and BCG vaccinated mice. No difference was observed for CD4+ and CD8+ T cells with a T effector memory cell phenotype (CD44^hi^CD62L^lo^) between Msm Δ*espG*_3_::*mtp64* and BCG.

To address whether the Δ*espG*_3_ deletion or MPT64 expression in Msm are required for the effector T cell polarization toward Tc1 and Tc17 cells, we analyzed effector T cell responses in mice that received Msm WT, Msm Δ*espG*_3_ or Msm Δ*espG*_3_::*mtp64* vaccination before Mav infection. Msm Δ*espG*_3_::*mtp64*-vaccinated mice showed the highest frequencies of IL-17-producing CD4+ (Th17) and CD8+ (Tc17) T cells (Supplementary Figure 2B).

Taken together our results suggest that even if all Msm vaccine strains mediated protection to Mav infection, only the EspG_3_ disrupted Msm strain overexpressing the MPT64 antigen favored polarization of Mav-specific T cell responses toward CD4+ Th17 and CD8+ Tc17 T cells.

### Vaccination With Msm *ΔespG_3_*::*mtp64* Elicits T Cells With Therapeutic Potential in Mav Infection

**Figure 4:**
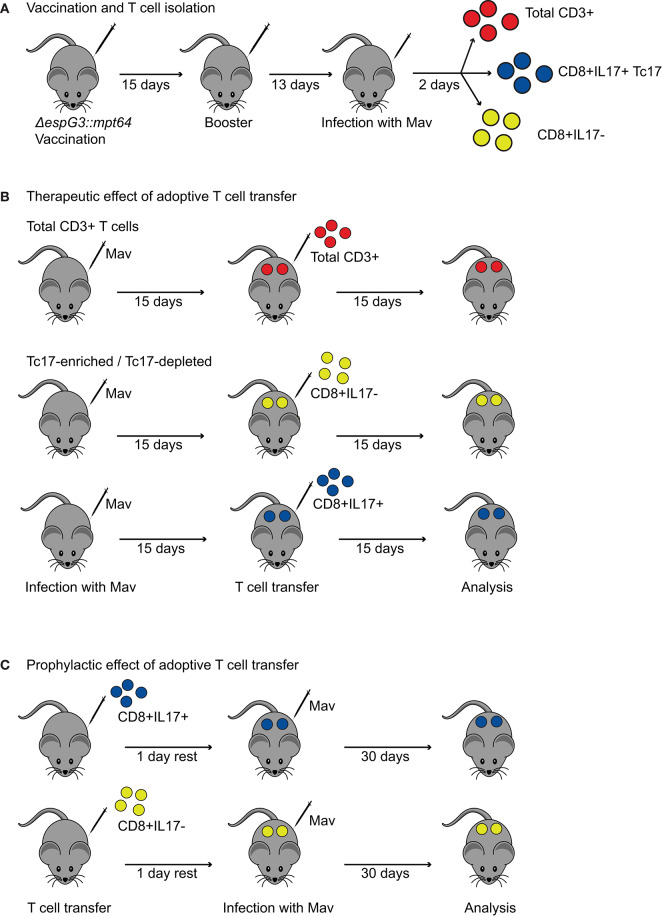
Procedures for adoptive transfer of T cells from Msm Δ*espG*_3_::*mpt64*-vaccinated mice. **(A)** Vaccination and T cell isolation for adoptive transfer. Mice received two subcutaneous vaccinations with Msm Δ*espG*_3_::*mpt64* or sham-vaccination with a 15-day interval. Twenty-eight days after the first vaccination, mice were challenged with Mav. On day 30 after the first vaccination, spleens were harvested, and total CD3+ or Tc17-enriched and Tc17-depleted CD8+ T cells isolated for adoptive transfer. **(B)** Experimental setup to study the therapeutic effect of adoptive T cell transfer on Mav infection. C57BL/6 mice were infected for 15 days before adoptive T cell transfer. Total CD3+ T cells or Tc17-enriched as well as Tc17-depleted CD8+ T cells from Δ*espG*_3_::*mpt64*-vaccinated mice were transferred. Bacterial load and T cell responses were analyzed 15 days post-adoptive T cell transfer. **(C)** Experimental setup to study the prophylactic effect of adoptive T cell transfer on Mav infection. Tc17-enriched as well as Tc17-depleted CD8+ T cells from Δ*espG*_3_::*mpt64*-vaccinated mice were injected into naïve C57BL/6 mice 1 day prior to infection with Mav. Effects of adoptive transfer on bacterial load and T cell responses in mice were analyzed 30 days post Mav infection.

**Figure 5:**
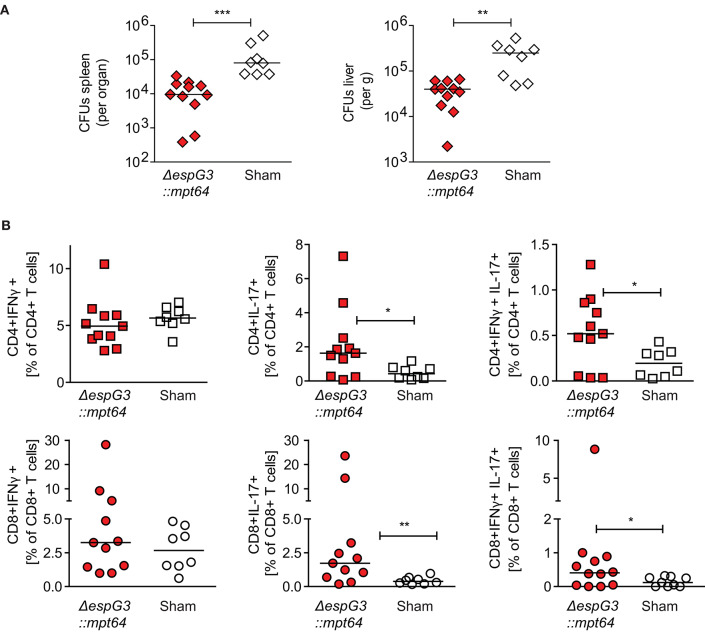
CD3+ T cells from Msm Δ*espG*_3_::*mpt64-*vaccinated mice have a therapeutic effect on Mav infection. Total CD3+ T cells were isolated from Msm Δ*espG*_3_::*mpt64* or sham-vaccinated mice and adoptively transferred to Mav-infected mice as described in Figures 4A,B. Liver and spleens of mice were harvested 15 days post-transfer to analyze bacterial load and Mav-specific T cell functions. **(A)** Mav CFUs were quantified in spleen (left) and liver (right). **(B)** Mav-specific CD4+ and CD8+ T cell effector cytokine production was quantified by flow cytometry after *in vitro* re-stimulation of splenocytes with Mav. Frequencies of IFNγ-(left), IL-17-(middle), and IFNγ/IL-17-producing (right) CD4+ and CD8+ T cells were analyzed as % of CD4+ (top row) or CD8+ (lower row) T cells. Cells were gated as described in Supplementary Figure 3. Graphs show the combined results from two individual adoptive transfer experiments with four to six mice in each group. *p < 0.05, **p < 0.01, ***p < 0.0003 by Mann–Whitney *U*-test.

Since we found that vaccination with Msm Δ*espG*_3_::*mtp64* conferred protection to Mav infection and polarization toward IL-17-producing effector T cells, we wanted to demonstrate that the protective effect against Mav infection originates from the T cell compartment. Therefore, we purified CD3+ T cells from Msm Δ*espG*_3_::*mtp64-* or sham-vaccinated vaccinated mice and adoptively transferred them into unvaccinated mice that were pre-infected for 15 days with Mav strain 104 (Figures 4A,B). Thirty days after adoptive CD3+ T cell transfer from vaccinated mice, organ bacterial load, and effector T cell responses were analyzed in the recipient mice (Figure 5). Bacterial load in spleen and liver of mice that received adoptive transfer of CD3+ T cells from Msm Δ*espG*_3_::*mtp64*-vaccinated mice was significantly reduced compared to mice that received CD3+ T cells from sham-vaccinated mice (Figure 5A). These results demonstrate that effector T cells from Msm Δ*espG*_3_::*mtp64*-vaccinated mice have a therapeutic potential and convey to protection from Mav infection in the recipient mice.

We further analyzed the proportions of IFNγ- or IL-17-producing Mav-specific effector T cells in spleens of the recipient mice. Adoptive transfer of CD3+ T cells from Msm Δ*espG*_3_::*mpt64*- or sham-vaccinated mice did not change the total T cell frequencies of IFNγ-producing CD4+ Th1 or CD8+ Tc1 cells (Figure 5B). However, the frequencies of IL-17 single-producing or IL-17/IFNγ double-producing CD4+ Th17 and CD8+ Tc17 cells were significantly increased in mice receiving CD3+ T cells from Msm*-*vaccinated mice compared to mice receiving T cells from sham-vaccinated mice (Figure 5B). These results suggest that T cells confer the protective effect of Msm vaccination against Mav infection. It has to be elucidated if the significantly increased frequencies of IL-17-producing Th17 and Tc17 that we found in Msm-vaccinated mice contribute to the protective effect.

### Tc17 Cells Have a Therapeutic Effect in Mav Infection

The most striking difference between BCG and Msm Δ*espG*_3_::*mtp64* vaccination was a shift from an IFNγ-dominated (Th1/Tc1) effector T cell response in BCG-vaccinated mice toward an effector response with increased frequencies of IL-17-producing CD4+ Th17 and CD8+ Tc17 effector T cells in Msm Δ*espG*_3_::*mtp64*-vaccinated mice. Since very little is known about the effect of CD8+ Tc17 cells in mycobacterial infections, we set out to investigate if Tc17 cells could have a protective role during Mav infection. For this, we adoptively transferred Tc17-enriched or -depleted CD8+ T cell fractions from Msm Δ*espG*_3_::*mtp64*-vaccinated mice into unvaccinated recipient mice. We tested if transfer of Tc17 cells protects mice when administered before the Mav infection is established (prophylactic effect) as well as the effect of administering Tc17 effector cells to an ongoing Mav infection (therapeutic effect).

CD8+ T cells were isolated from spleens of Msm Δ*espG*_3_::*mtp64*-vaccinated mice and separated into fractions enriched or depleted for CD8+ Tc17 cells. The CD8+IL-17+ T cell fraction contained >10% IL-17+ T cells, while the CD8+IL-17– fraction was devoid of IL-17+ T cells (Supplementary Figure 6). The Tc17-enriched and Tc17-depleted cell fractions were then adoptively transferred to naïve mice prior to challenge with Mav (prophylactic) or 15 days post-infection with Mav (therapeutic) (experimental procedures shown in Figure 4). Due to the incomplete enrichment of Tc17 cells, recipient mice received less Tc17 cells than estimated.

**Figure 6:**
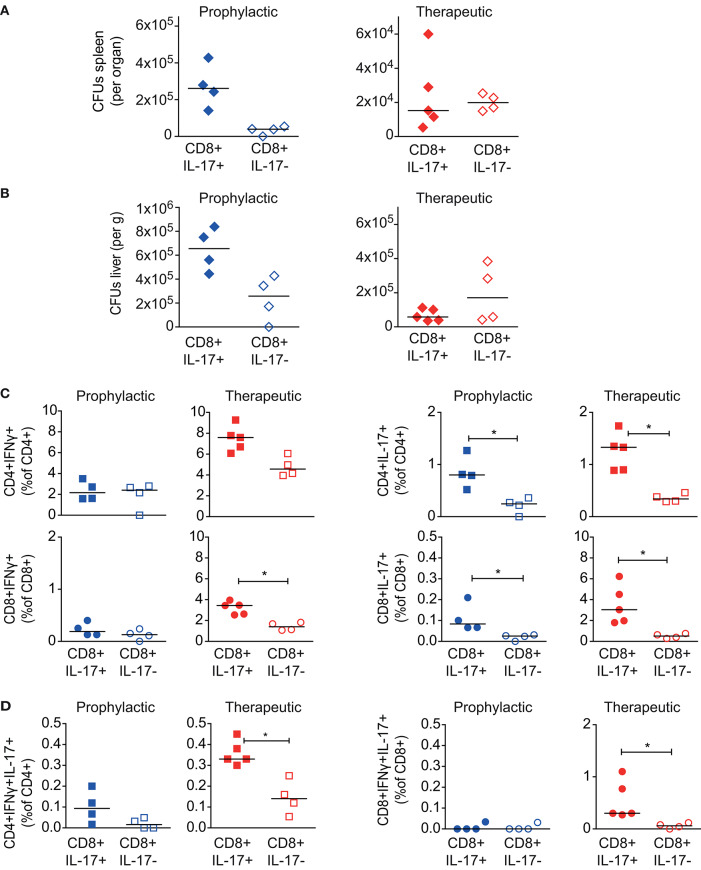
Effect of CD8+ Tc17 cells from Msm Δ*espG*_3_::*mpt64-*vaccinated on Mav infection. Tc17-enriched (CD8+IL-17+) or Tc17-depleted (CD8+IL-17–) CD8+ T cells were isolated from Msm Δ*espG*_3_::*mpt64*-vaccinated mice. Cells were adoptively transferred in a therapeutic approach (blue symbols) to mice pre-infected with Mav or in a prophylactic (red symbols) approach before infection of mice as described in Figure 4. Liver and spleens of mice were harvested in both approaches on day 30 post Mav-infection for analysis of bacterial load and effector T cell functions. **(A,B)** Mav CFUs were quantified in spleen **(A)** and liver **(B)** of mice. **(C,D)** Splenocytes were re-stimulated with Mav overnight and frequencies of IFNγ or IL-17 effector cytokine-producing (**C**, IFNγ left, IL-17 right graphs) or IFNγ/IL-17 double-producing **(D)** CD4+ and CD8+ T cells were quantified by flow cytometry (% of CD4+ or CD8+ T cells). T cells were gated as described in Supplementary Figure 3. Two individual adoptive transfer experiments with four or five mice in each group were performed. Graphs show results from one representative experiment. *p < 0.05 by Mann–Whitney *U*-test. Purity analysis of adoptively transferred cells is shown in Supplementary Figure 6.

Bacterial load and effector T cell responses in recipient mice were analyzed in both setups 30 days after adoptive T cell transfer. Adoptive transfer of Tc17-enriched or Tc-17-depleted CD8+ T cells did not significantly reduce organ bacterial loads in spleen (Figure 6A) or liver (Figure 6B) of the recipient mice. This was the case for the prophylactic as well as the therapeutic experimental setup.

Finally, we analyzed the Mav-specific effector T cell composition in in recipient mice 30 days post transfer of Tc17-enriched or -depleted cells. Since we did not trace the adoptively transferred T cells, we could not distinguish if these cells originated from the donor or recipient mice. In the prophylactic setup, we observed no differences in the IFNγ-producing CD4+ Tc1 or CD8+ Tc1 cells in mice receiving Tc17-enriched or Tc17-depleted cells (Figure 6C, left). However, upon therapeutic transfer to mice infected for 15 days with Mav, we found higher frequencies of total IFNγ-producing CD4+ and CD8+ T cells if mice received Tc17-enriched cells (significant for CD8+IFNγ+, Figure 6C, left). Moreover, transfer of Tc17-enriched cells was found superior in activating Mav-specific IL-17 responses in recipient mice, both for prophylactic and therapeutic transfer (Figure 6C, right). Frequencies of both, CD4+ Th17 as well as CD8+ Tc17 cells were significantly increased in mice that that received Tc17-enriched cells. The frequencies of IFNγ/IL-17 double-producers were also found significantly increased after therapeutic transfer of Tc17-enriched cells (Figure 6D).

Taken together our results suggest that CD8+ Tc17 cells may have a therapeutic role in mouse Mav infection, although they do not seem to be required for protection. Further studies are needed to clarify the role of Tc17 cells in mycobacterial infections.

## Discussion

In the current study, we show that Msm and Msm mutants might function as a vaccine against Mav infection similar to vaccination with *M. bovis* BCG. The BCG vaccine is recognized as safe by the WHO, and in addition to prevent tuberculosis, the BCG vaccine has been used to treat bladder tumors for more than 30 years. Despite the success, there is a concern with toxicity of the BCG cancer treatment and a risk of disseminated BCG disease upon vaccination of immunocompromised individuals, which is contraindicated (40). The vaccine we investigated in this study is based on Msm, a non-pathogenic mycobacterium which is cleared quickly from human macrophages (41). In model systems lacking functional NK cells and T cells, Msm is well tolerated (40, 42). Recently, vaccination with a Msm mutant in *esx-3* was shown to protect mice against Mtb infection, demonstrating cross-reactivity and cross-protection between Msm and other mycobacteria (29). The Msm *esx-3* mutant was rapidly cleared from mice after intravenous infection with high numbers of bacteria, without any adverse effects in the animals (29). Despite a handful of cases with Msm infected individuals (43), the overall picture shows that Msm based vaccines would offer improved safety profiles compared to BCG.

A study by Sweeney et al. (29) showed that *esx-3* deletion in Msm leads to decreased virulence, enhanced induction of IL-12 and IFNγ, and a strong Th1 response. Reduced organ bacterial loads could result from restricted growth, e.g., from iron starvation resulting from ESX-3 deletion (28), or from improved bacterial clearance due to loss of ESX-3 secreted factors modulating inflammatory responses, which could improve antigen presentation. Our study is in favor of the latter scenario as we found significantly reduced bacterial load and reduced expression of CD86 in APCs infected with the Msm Δ*espG*_3_, which could indicate reduced inflammatory properties of the Δ*espG*_3_ mutant strain. This reduced virulence did not impair antigen-presentation capabilities of the Msm Δ*espG*_3_ mutant as we detected equal or increased presentation of ovalbumin model antigens to CD4+ and CD8+ T cells with Msm Δ*espG*_3_ when compared to wild-type Msm. *In vitro* antigen presentation and CD4+ and CD8+ T cell activation was not directly addressed by the Sweeney et al. (29).

We could show a protective effect of Msm Δ*espG*_3_::*mpt64* vaccination on subsequent Mav infection in mice, as organ bacterial loads were decreased compared to unvaccinated mice. The level of protection was found to be comparable to BCG vaccination, suggesting the presence of shared antigens in Msm, BCG, and Mav. Additional expression of the Mav protein MPT64 did not enhance the protective effect of the Msm vaccine on organ bacterial load. However, we only observed a differential development of Mav-specific effector T cell subsets in mice that received vaccination with the Msm strain that overexpressed the MPT64 antigen. BCG-vaccinated mice showed a Th1- and Tc1-dominated immune response with the majority of Mav-specific T cells producing IFNγ. In contrast, mice vaccinated with Msm Δ*espG*_3_::*mpt64* showed a bias toward IL-17-producing CD4+ Th17 and CD8+ Tc17 T cells. In addition, increased frequencies of polyfunctional CD4+ T cells producing IFNγ, IL-17 as well as TNFα were observed in Msm Δ*espG*_3_::*mpt64*-vaccinated mice. Overexpression of the Mav antigen MPT64 seemed to be required, as no Th17/Tc17 bias was observed in mice vaccinated with Msm WT or the Msm Δ*espG*_3_ mutant that did not express the MPT64 antigen. These results indicate that Th17 and Tc17 responses could be induced by expression and secretion of the MPT64 antigen, but more studies are needed to confirm if this is the case. Junqueira-Kipnis et al. (44) demonstrated that mice vaccinated with Msm Δ*esx3* overexpressing fusion protein (Ag85c, MPT51, and HspX) produced high frequencies of Th17 cells in the lungs on challenge with Mtb, accompanied with lower organ bacterial loads. In addition, Matsuyama et al. (45) showed that IL-17 production increased in CD4+ T cells upon infection with Mav, a minor population of CD8+ Tc17 cells expressing IL-17 was also detected. We also observed low levels of Tc17 cells in unvaccinated and BCG-vaccinated mice in our study. However, vaccination of mice with Msm Δ*espG*_3_::*mtp64* prior to Mav infection was found to increase the frequencies of IL-17-producing Th17, Tc17 as well as IFNγ/IL-17 double-producing CD4+ and CD8+ T cells. One explanation could be that a proinflammatory milieu (IL-6,IL-1β, IL-23, and IL-12) generated by the vaccine aided in the generation of IL-17-producing T cells (46). It might also be that Msm Δ*espG*_3_::*mtp64* induces apoptosis of APCs in the draining lymph nodes and thereby engages CD8+ cells. This has previously been shown for dendritic cells that take up apoptotic macrophages carrying mycobacterial antigens and subsequently trigger the proliferation of CD8+ cells via cross-presentation (47–49). Finally, it might also be that the vaccine causes a contact hypersensitivity-like reaction, which has been shown to engage CD8+ T cells with production of IFNγ and IL-17 (50, 51).

Most of the knowledge about IL-17 in Mtb pathogenesis comes from investigations on CD4+ Th17 cells (15) and γ/δ+ T cells (52). Protective effects of Th17 cells have also been highlighted in a vaccine model against pulmonary TB (12–14). Khader et al. (13) demonstrated that CD4+IL-17+ T cells from vaccinated mice upregulated chemokine production, neutrophil recruitment, and promoted tissue infiltration of IFNγ-producing CD4+ T cells, leading to restriction in mycobacterial growth. In our study, IL-17-producing T cells and polyfunctional CD4+ T cells (IFNγ+/IL-17+/TNFα+) could have a similar role by promoting cell infiltration leading to reduced bacterial growth. Moreover, a recent study showed that BCG or Mtb infection can prime fully differentiated IFNγ+/TNFα+ effector CD4+ memory T cells with lower lung homing capacity than H56/CAF01 subunit vaccination, which induces central memory T cells dominated by IL-2- and IL-17-producing CD4+ T cell subsets (53). It could be that the Msm Δ*espG*_3_::*mtp64* vaccine resembles the H56/CAF01 subunit vaccine in yielding memory T cells with superior homing capacity to infected tissues compared to BCG. Our finding that Msm Δ*espG*_3_::*mtp64* induced more CD4+ central memory T cells than BCG supports this hypothesis, although it needs to be experimentally confirmed.

It is currently unknown if Tc17 cells have a protective role in mycobacterial infections. Interestingly, a recent study showed that infants vaccinated with BCG strain VPM1002 had high proportions of Tc17 cells (20). In Mav infection, there has only been observed small populations of Tc17 cells and no specific function has been tested (45). The role of Tc17 cells is better investigated in other diseases like cancer, fungal and viral infections (29, 54, 55). Tumor studies have shown that Tc17 cells, though less cytotoxic, are highly plastic and actively convert to cytotoxic IFNγ/IL-17 double producers in the presence of IL-12 (55, 56). Satoh et al. (57) have suggested that in the presence of IL-12, suppression of SOCS3 in Tc17 cells permits the induction of both IL-17 and IFNγ, yielding cytotoxic Tc17 cells (57). In our study, we tested for a direct role of Tc17 cells in Mav infection. We saw no protective effect from prophylactic transfer of Tc17 cells from Msm Δ*espG*_3_::*mtp64* vaccinated mice to naïve mice prior to Mav infection. We contemplate several reasons as an explanation for the failed protective effect of Tc17 cell transfer. First, Tc17 cells may need an adequate inflammatory milieu to survive and expand, which is not provided by naïve mice. Cells could thus be dead before mice are infected with Mav one day after adoptive transfer. In addition, the Tc17 cells could only be enriched to >10% of CD8+IL-17+ T cells in this study. Alternatively, we speculate that Tc17 cells, when reaching the immunological niche, may turn anergic due to low density of stimuli (58–61). We did not trace the adoptively transferred Tc17-enriched and Tc17-depleted T cells in our study and were thus unable to distinguish if the effector T cell responses in the recipient mice originated from the adoptively transferred cells or from immune cells of the recipient mice. The experiments are thus to be regarded as initial proof of concept studies for future follow-up, for example with mice expressing different CD45 isoforms. Tracking of Tc17-enriched donor cells could then allow analysis of survival, tissue homing and plasticity of the transferred cells and answer the question if the observed effect is due to a direct effector function of the transferred cells or if the transferred cells shape effector responses of the immune cells in the recipient mouse. In our model system, Tc17 cells were transferred into naïve mice without prior irradiation. It has been demonstrated that transfer of both BCG induced immunity and antitumor efficacy of Tc cells require depletion of immune cells through irradiation (62, 63). However, Sweeney et al. (29) observed that memory CD4+ cells from Msm Δ*esx3*::*Mtbesx-3* vaccinated mice were able to provide protection of naïve mice without irradiation prior to transfer. On the other hand, when the Tc17 cells were injected into mice pre-infected with Mav (therapeutically), we observed increased protection with lower organ bacterial loads, as well as higher frequencies of CD8+IFNγ+, CD8+IL-17+, and CD8+IFNγ+IL-17+ T cells when compared to mice receiving Tc17 as a prophylactic measure. This suggests that Tc17 cells may require an inflammatory milieu to provide adequate protection. It could be that the Tc17-enriched cells were terminally differentiated at the time of the transfer and could not be re-activated to become effector cells upon a later challenge with Mav in the prophylactic setting. Lastly, it might be that adoptive transfer of an isolated T cell subset such as CD8+ Tc17 cells is not enough to protect the mice from infection and that other T cell subsets such as CD4+ Th17 cells or IFNγ-producing Th1 and Tc1 cells are required for protection. This is supported by our observation that transfer of total CD4+ and CD8+ T cells, but not Tc17-enriched T cells alone, from Msm Δ*espG*_3_::*mtp64*-vaccinated mice mediated protection from Mav infection.

In this study, we have demonstrated the potential of Msm Δ*espG*_3_::*mtp64* as a safe vaccine that can be further developed against mycobacterial infections. Msm Δ*espG*_3_::*mtp64* vaccination elicited a higher proportion of IL-17-producing Th17 and Tc17 in comparison to BCG vaccination, which elicited a Th1/Tc1-dominated immune response. In this study, we used *i.p*. infection of mice as infection model, which leads to reproducible systemic infection of mice, but is of less physiological relevance than other routes of infection. Future studies are needed to substantiate the importance of Th17/Tc17 cells in protecting against Mav infection, e.g., with different and more physiological routes of Mav infection, by tracing the Tc17 cells in the recipient mice or by neutralizing IL-17 antibodies, and to establish their function and possible protective capabilities.

## Data Availability Statement

The datasets generated for this study are available on request to the corresponding author.

## Ethics Statement

The animal study was reviewed and approved by the Norwegian National Animal Research Authorities.

## Author Contributions

NK, MH, MS, and TF: conceived and designed the experiments and wrote the manuscript. NK and MH: performed the experiments.

## Conflict of Interest

The authors declare that the research was conducted in the absence of any commercial or financial relationships that could be construed as a potential conflict of interest.

## Abbreviations

APC: Antigen-presenting cell
BCG: *Mycobacterium bovis* bacillus Calmette–Guérin
BMDC: Bone marrow-derived dendritic cell
BMDM: Bone marrow-derived macrophage
CFU: colony forming unit
IFN: interferon
IL: interleukin
Mav: *Mycobacterium avium*
MOI: multiplicity of infection
MHC: major histocompatibility complex
Msm: *Mycobacterium smegmatis*
Mtb: *Mycobacterium tuberculosis*
NTM: non-tuberculous mycobacteria
OVA: ovalbumin
PBS: Phosphate-buffered saline
TB: Tuberculosis
Tc: CD8+ cytotoxic T lymphocyte
Th: CD4+ helper T lymphocyte
TNFα: tumor necrosis factor α
WT: Wild-type.
