# Supplementary material for: Mycobacterium smegmatis Vaccine Vector Elicits CD4+ Th17 and CD8+ Tc17 T Cells With Therapeutic Potential to Infections With Mycobacterium avium
Source: Front Immunol. 2020 Jun 9;11:1116. doi: 10.3389/fimmu.2020.01116 (PMC7296097; doi:10.3389/fimmu.2020.01116)
Supplement: Supplementary file 1 [file Data_Sheet_1.pdf]

Supplementary Figure 1

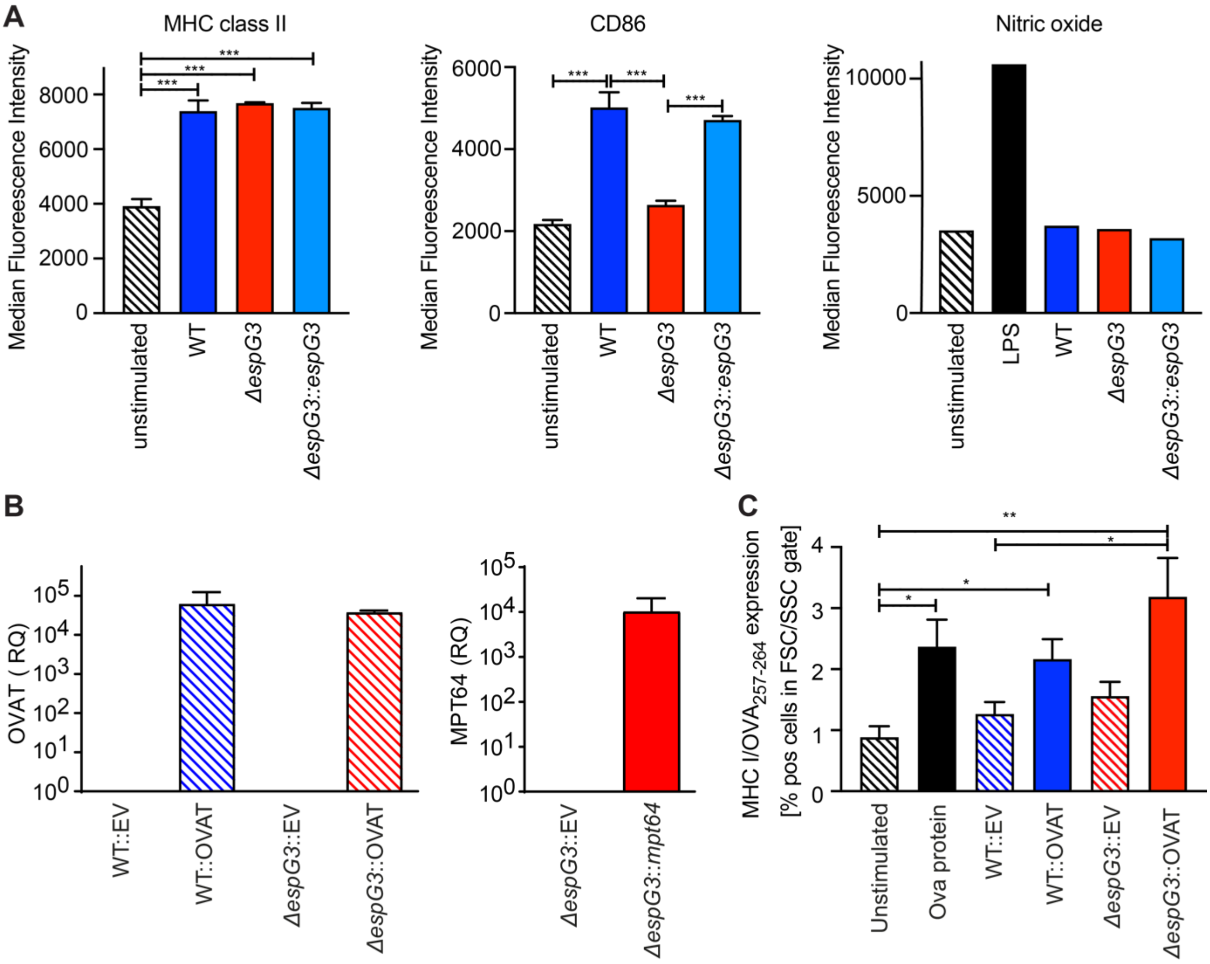

Figure S1. Supplementary data supporting main Figure 1.

(A) Costimulatory molecule expression and nitric oxide production in Msm-infected macrophages. BMDMs were infected for 24h with Msm wild-type (WT), Msm *ΔespG3* or reconstituted Msm *ΔespG3::espG3*. Surface expression of MHC class II (I-Ab, left graph) and CD86 (center graph) as well as nitric oxide production (DAF-FM diacetate staining, right graph) was analyzed by flow cytometry. Experiments for MHC class II and CD86 expression were analyzed from three, nitric oxide production from two individually stimulated samples (no error bars shown for nitric oxide production).

(B) OVA and MPT64 antigen expression analysis in Msm. Msm WT or Msm *ΔespG3* were transformed with truncated ovalbumin (OVAT) or Mav antigen MPT64 (only msm *ΔespG3::mpt64*) in the destination vector pDE43. Transfection with the empty destination vector (EV) was performed as control. Relative mRNA expression of OVAT (left graph) or MPT64 (right graph) were analyzed by qPCR. All bacterial strains were grown to exponential phase before mRNA isolation. qPCR data was normalized to expression of RNA polymerase sigma factor SigA (reference for gene expression). Mean + SEM from two independent experiments are shown.

(C) MHC class I antigen-presentation from Msm-infected antigen-presenting cells. BMDCs were infected with Msm WT or Msm *ΔespG3*, either overexpressing truncated ovalbumin protein (WT::OVAT and *ΔespG3*::OVAT) or transformed with the empty destination vector (EV) pDE43 alone (WT::EV and *ΔespG3*::OVAT). BMDCs stimulated with OVA protein (225 µM) served as positive control, unstimulated BMDCs as negative control. 24h post infection, MHC class I presentation of the antigenic $OVA_{257-264}$ peptide was assessed by flow cytometry (H-2Kb/SIINFEKL antibody staining). Results in all graphs are displayed as means + SEM; statistical analyses were performeded using one-way ANOVA with Tukey post-test; significance level: *p < 0.05, **p < 0.01, ***p < 0.001.

Supplementary Figure 2

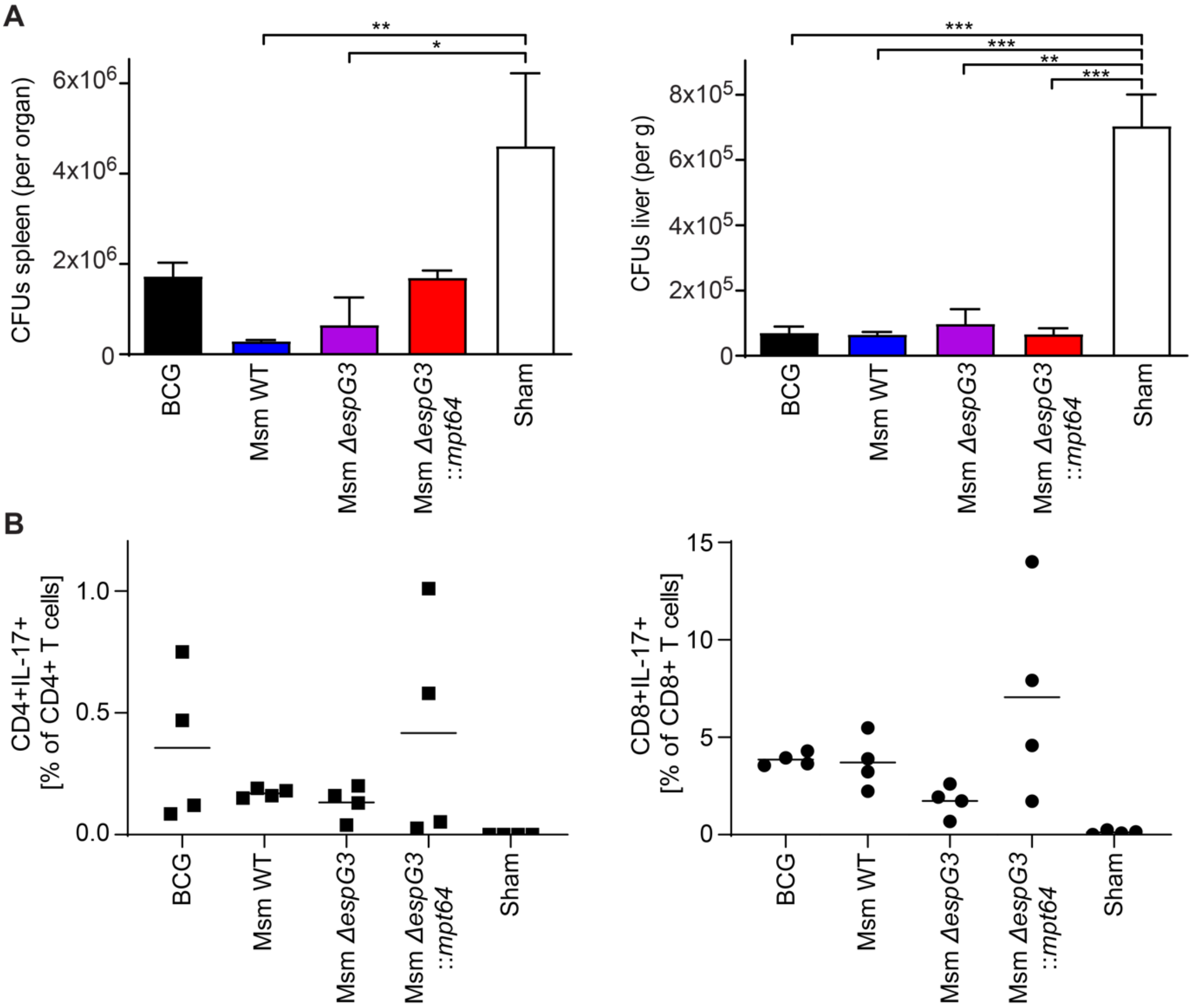

Figure S2. Effect of vaccination with different Msm strains on Mav infection.
Mice were vaccinated with BCG, Msm WT, Msm *Δespg3* or Msm *Δespg3::mpt64* and challenged with Mav as described in Figure 2A. Sham-vaccinated mice served as controls. On day 30 post Mav challenge, livers and spleens were harvested. (A) Bacterial load (CFUs) was quantified in spleen (left) and liver (right). Shown are the results from one out of two vaccination experiments with four mice per group; bars represent mean + SEM. Statistical significance was determined using repeated measures one-way ANOVA with Tukey`s post-test; significance levels: *$p < 0.05$; **$p < 0.01$; ***$p<0.0001$.
(B) Mav-specific IL-17 production from CD4+ T cells (left) and CD8+ T cells (right) was was quantified from *in vitro* re-stimulated splenocytes. Shown are the results from one out of two vaccination experiments with four mice per group. Differences between the vaccination groups were not statistically significant (one-way ANOVA with Tukey`s post-test).

Supplementary Figure 3

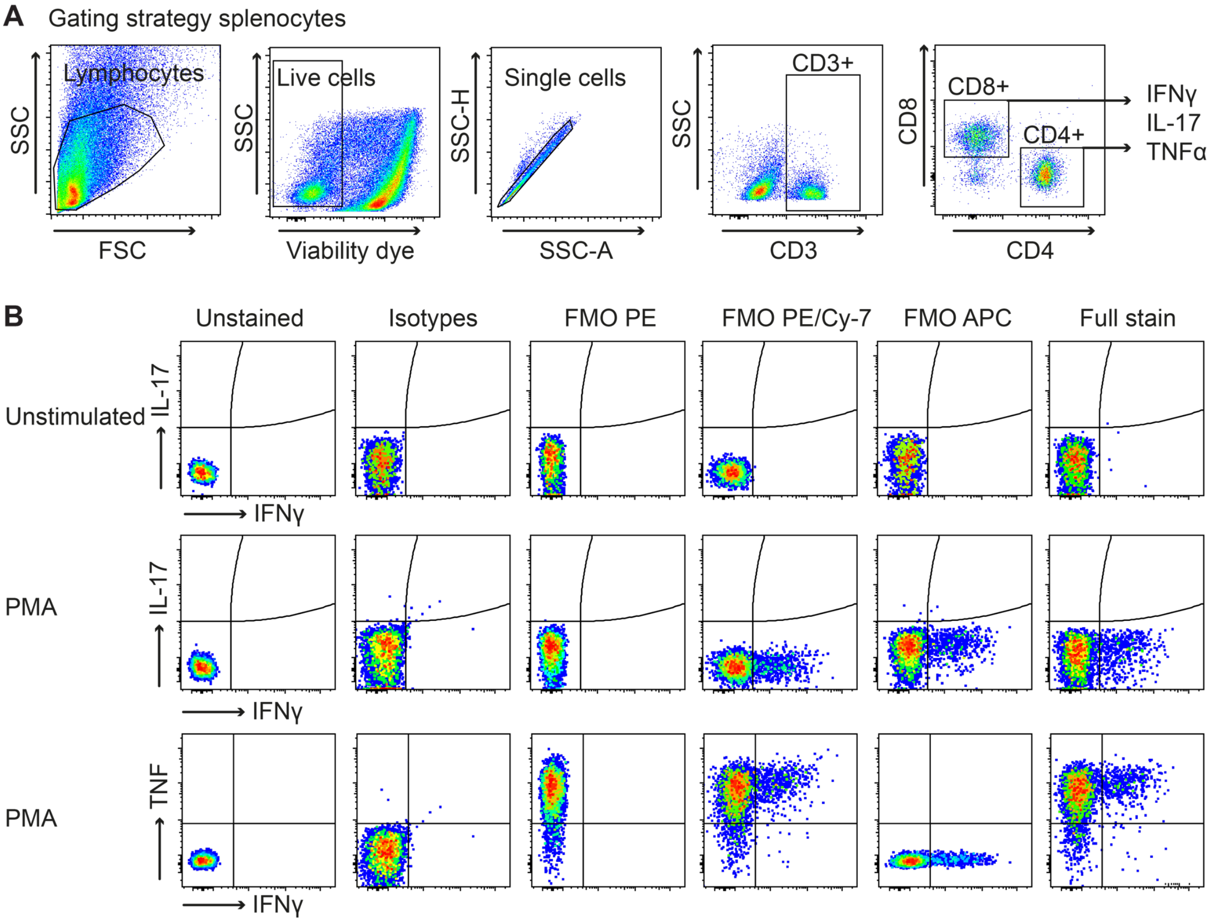

Figure S3. Flow cytometric gating strategy and staining controls for cytokine analysis.
(A) The flow cytometric gating strategy to identify viable CD4+ and CD8+ T cells from splenocytes is exemplified.
(B) Fluorescence Minus One (FMO) controls for flow cytometric cytokine analysis. Pooled splenocytes from mice in all vaccination groups were left unstimulated or treated for 4h with PMA and protein transport inhibitor cocktail before analysis. CD4+ and CD8+ T cells were identified as described in (A). Flourescence minus one (FMO) controls were performed for gating of IFNγ+, TNFα+ and IL-17+ T cells. Shown are representative examples for gating of CD8+ T cells.

Supplementary Figure 4

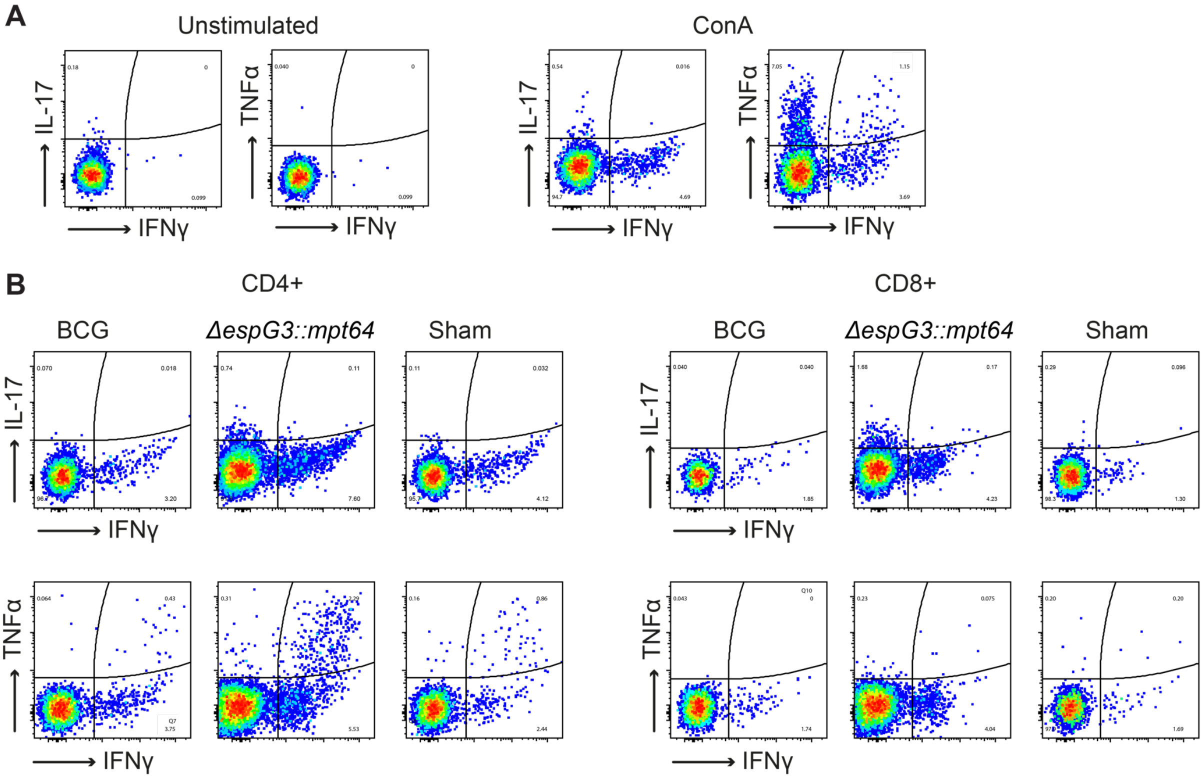

Figure S4. Staining examples for flow cytometric analysis of CD4+ and CD8+ T cell cytokine production. Splenocytes were isolated from vaccinated mice that have been challenged with Mav as described in Figure 2A.

(A) Positive and negative controls for cytokine analysis production. Splenocytes were left unstimulated (negative control) or treated overnight with ConA (positive control). Examples from CD4+ T cells of a *Δespg3::mpt64* vaccinated mouse.

(B) Analysis examples of Mav-specific effector cytokine production from CD4+ and CD8+ T cells. Splenocytes from BCG, Msm *Δespg3::mpt64* or sham-vaccinated mice were re-stimulated overnight with Mav, before IFNγ, TNFα and IL-17 production were analyzed by intracellular flow cytometry. Samples were treated with protein transport inhibitor for the last 4h of stimulation. CD4+ (left) and CD8+ T cells (right) were identified as described in Supplementary Figure S3.

Supplementary Figure 5

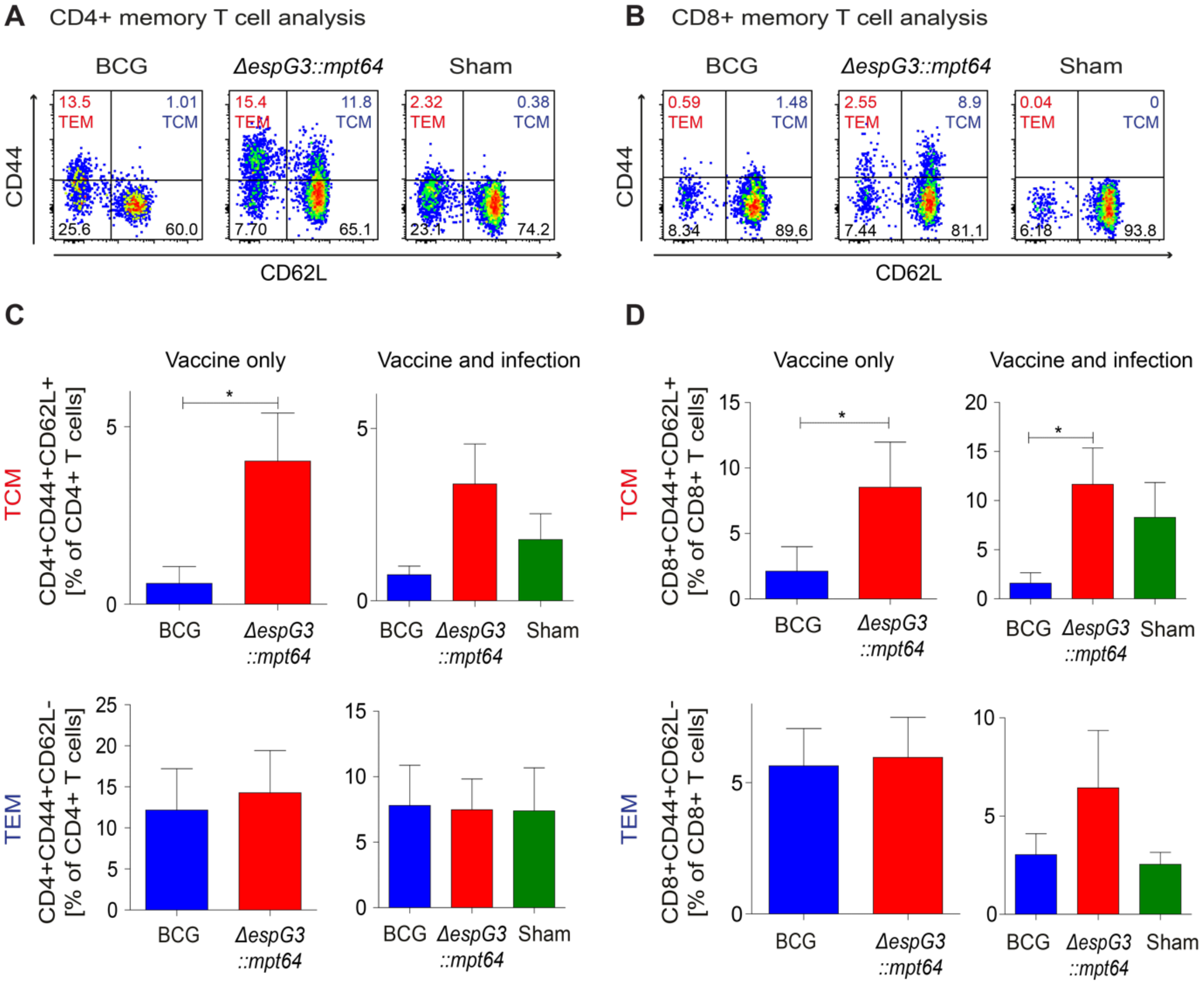

Figure S5. Memory T cell responses in vaccinated mice.
CD4+ and CD8+ T cells of vaccinated mice vaccinated were analyzed for central memory (TCM) or effector memory (TEM) T cell marker expression. Analysis was either performed directly after vaccination or after challenge with Mav for 30 days as described in Figure 2A.
(A,B) Examples for flow cytometric TCM (CD44+CD62L+) and TEM (CD44+CD62L-) analysis of CD4+ (A) and CD8+ (B) T cells. CD4+ and CD8+ T cells were identified as described in supplementary Figure S3. Shown are examples from uninfected mice that received BCG, *Δespg3::mpt64* or sham vaccination.
(C,D) Quantification of CD4+ (C) and CD8+ (D) T cells with a TCM (CD44+CD62L+, upper row) or TEM (CD44+CD62L-, lower row) phenotype. Frequencies of TCM and TEM were analyzed from mice that received vaccine only (no Mav challenge, left graphs) or from mice that received vaccine and were subsequently challenged with Mav for 30 days (right graphs). Results are displayed as % of CD4+ or CD8+ T cells. Vaccine only graphs: Bars represent means + SEM from two experiments with four mice in each group. Significance levels were analyzed using paired t-test; *p<0.05. Vaccine and Mav infection experiments: Bars represent means + SEM from three experiments with four mice in each group. Significance levels between all groups were analyzed using repeated measures one-way ANOVA with Tukey post-test; *p<0.05.

Supplementary Figure 6

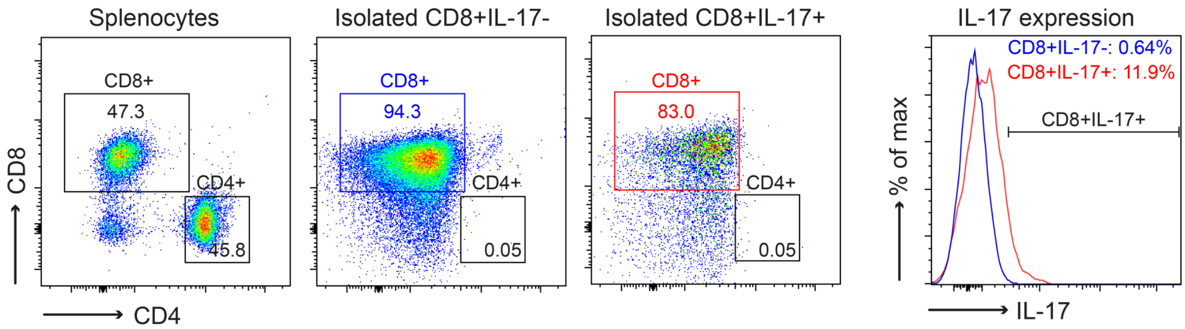

Figure S6. Analysis of T cells used for adoptive transfer experiments.
T cells for adoptive transfer experiments were isolated from mice that have been vaccinated as described in Figure 4A. Purity of T cell preparations was analyzed by flow cytometry. Example shows isolation of CD8+IL-17- and CD8+IL-17+ T cells.
